# Parenteral exposure to pesticides and occurence of congenital malformations: hospital-based case–control study

**DOI:** 10.1186/s12887-016-0667-x

**Published:** 2016-08-12

**Authors:** Marly Eliane Ueker, Vivianne Monteiro Silva, Gisele Pedroso Moi, Wanderley Antonio Pignati, Ines Echenique Mattos, Ageo Mário Cândido Silva

**Affiliations:** 1 National School of Public Health (ENSP/FIOCRUZ), Rio de Janeiro, Brazil; 2 Institute of Collective Health, Federal University of Mato Grosso (UFMT), Av. Fernando Corrêa, 2367. CCBSIII Block, 2nd floor. Boa Esperança, 78060-900 Cuiabá, MT Brazil; 3 University Center of Várzea Grande (UNIVAG/MT), Várzea Grande, Brazil

## Abstract

**Background:**

Most fetal defects are associated with genetic and environmental causes, among them, exposure of pregnant women to intensive pesticide use. Agribusiness is the economic basis of the state of Mato Grosso, the largest consumer of pesticides of all Brazilian states. The objective of this study was to investigate the association between past parental exposure to pesticides and the occurrence of congenital malformations in children in Mato Grosso, Brazil.

**Methods:**

This hospital-based case–control study was conducted in Cuiabá, the capital of Mato Grosso, from March to October 2011. Data was collected in all public, private, and health plan referral hospitals that provide care for pregnant women in the state of Mato Grosso and were situated in Cuiabá. Cases were children under 5 years of age with congenital malformations classified in Chapter XVIII of the International Classification of Diseases-10 and controls were children within the same age range, without congenital malformations, treated at the same hospitals. Malformation-related data was obtained from the patients’ medical records. Socioeconomic data and information about parental exposure to pesticides were obtained in an interview with the mother using a standardized questionnaire. We conducted multivariate logistic regression to assess the relation between parent report of past pesticide use and congenital malformations. We also assessed effect modification to verify whether low maternal education level modified the association between exposure and our outcome.

**Results:**

We observed positive effect modification of the association of paternal past exposure to pesticide and congenital malformation in the offspring by maternal education for mothers with low educational level (OR = 8.40, 95 % CI 2.17–32.52), father’s work related to farming (OR = 4.65, 95 % CI 1.03–20.98) and paternal past exposure to pesticides (OR = 4.15, 95 % CI 1.24–13.66).

**Conclusion:**

These findings provide further evidence that paternal exposure to pesticides, especially when associated with a low maternal education level, may be related to higher rates of fetal malformation in Mato Grosso, Brazil.

## Background

Roughly 3 to 5 % of live born children in the Latin America and other world regions have some sort of congenital malformation [1]. In Brazil, the South, Northeast, and Midwest regions have the highest prevalence of congenital malformations among live births [2], and these conditions are an important cause of infant mortality in developed countries [1]. Congenital malformations are related to many factors [3] including use of teratogenic medications in pregnancy [4], the development of viral infections when pregnant [5], genetic alterations and environmental factors. Pesticides are among some of the environmental factors that have been linked previously to malformations [6, 7].

Epidemiological studies have found a higher prevalence of congenital malformations in children born to mothers living in regions with high pesticide use [8–10]. Most pesticides are endocrine disruptors, having mutagenic, teratogenic and genotoxic action [11–13]. Thulstrup & Bonde (2006) [14] systematically reviewed 26 epidemiological studies published between 1966 and 2004 to assess the association of maternal occupational exposures during pregnancy and risk of some malformations. Ten of these studies included pesticide exposure-related farm work. Maternal farming activities were associated with neural tube defects, cleft lip, cleft palate and congenital heart disease in offspring [14].

González-Alzaga et al. (2015) [10] found a significant relationship between fetal teratogenic effects and maternal exposure to some pesticides that are widely used in the Midwest region of Brazil, where this study was conducted. Agribusiness is the economic basis of the state of Mato Grosso, the greatest consumer of pesticides among Brazilian states [15]. In 2009 the country used roughly 3.7 l of pesticides per inhabitant, while the state of Mato Grosso used 34.1 l per inhabitant [15]. According to the National Syndicate of the Industry for Defense of the Agricultural Product [16], glyphosate, an organophosphate, is the most consumed pesticide in the country, accounting for almost half the volume of all active ingredients marketed in Brazil, being the state of Mato Grosso the largest consumer of this product [15]. The association between glyphosate exposure and occurrence of fetal malformations was related in the literature [17].

Currently, there is only one study conducted in Mato Grosso, Brazil that analyzed the possible influence of pesticides on fetal malformation [7]. However, as it is an ecological study, it is not possible to extrapolate its results to the individual level [18].

The mechanisms responsible for the toxicity of pesticides on human fetal development have not yet been elucidated [19]. However, among the main findings are genotoxicity and mutagenicity promoted by organophosphates that were evidenced by studies in vitro [20], *in animals* [21] and *in humans* [22].

Given the high use of pesticides in this particular area and the scarcity of local investigations, the objective of this study was to investigate the association between past exposure of parents to pesticides and the occurrence of congenital malformations in children born in the State of Mato Grosso, Brazil.

## Methods

A hospital-based case–control study was conducted in Cuiabá, Mato Grosso, from March to October 2011 in four referral institutions that provide care to congenitally malformed children in the state [23]. The four institutions included public (Unified Healthcare System, SUS), private, and health-plan hospitals.

### Ascertainment of cases and controls

The cases consisted of children aged less than 5 years attending the abovementioned referral institutions whose main diagnoses in the medical records were congenital malformations classified in Chapter XVIII of the Tenth International Classifications of Diseases (ICD-10) [24]. The criteria for diagnosing a congenital malformation were based on the identification of structural or functional morphological changes present at birth or identified later by clinical examinations, laboratory tests, imaging techniques, and/or surgeries [25]. The controls consisted of children aged less than 5 years with other diagnoses attending the referral pediatric services of the same institutions due to respiratory diseases, infectious diseases, disorders of the perinatal period and endocrine and metabolic diseases. All children recruited during the study period were residents of Mato Grosso State. Cases and controls were matched by sex; for each case, a correspondent control was selected. Exclusion criteria included having a twin, being Ameridian, being born to a mother aged less than 18 years, and having incomplete information. This criteria was used for minimizing the influence of these characteristics on congenital malformations [26–28]. Sample size was calculated assuming a type I error of 5 %, test power of 80 %, two controls per case, maximum expected frequency of control exposure of 20 %, and a minimum odds ratio of 2.0. Hence, the sample should consist of 411 individuals, 137 cases and 274 controls. All mothers who agreed to join the study after the objectives were explained signed an informed consent form. The project was approved by the Research Ethics Committee of the University Hospital Júlio Müller under protocol number 935CEP/-HUJM/2011.

### Data collection and study variables

Data regarding parental exposure to pesticides was collected in an interview with the mother, based on a structured questionnaire designed for the study and clinical data was compiled from the hospital records of cases and controls. The variables selected for the study were those most cited in the literature as possible causes of malformations and included maternal smoking, maternal folic acid intake, maternal infections, maternal nutritional status, previous abortions, alcohol and drug use, medication use and immunization.

Maternal pesticide exposure period was defined as the 3 months that preceded conception and the first 3 months of pregnancy, a period where the risk of congenital malformations due to maternal exposure to environmental toxins is greatest. [9] Paternal pesticide exposure period was defined as the 12 months that preceded conception [29].

Socioeconomic and pregnancy-related data was collected with the instrument used for this purpose in the Collaborative Latin American Study on Congenital Malformations (CLASCM) [30].

We conducted a pilot test with 20 mothers with congenitally malformed children and 40 mothers with non-congenitally malformed children at the Pediatric Outpatient Clinic of the University Hospital Júlio Muller, in Cuiabá in order to evaluate the performance of the study questionnaire, before the beginning of the interviews. Mothers were interviewed either at the outpatient clinics or by their children’s bedside when the children were hospitalized. The interviewers were undergraduate students of the collective health program of the Institute of Collective Health of the Federal University of Mato Grosso, duly trained for data collection.

### Statistical analysis

We conducted univariate analysis, stratified and logistic regression using as dependent variable the presence or absence of congenital malformations. Congenital malformations were aggregated as a group for analysis instead of being analyzed as separate defects due to the inadequate numbers of specific phenotypes. Association measurements were estimated by the odds ratio (OR) with 95 % confidence limits [31]. We used Mantel-Haenszel chi-square test to evaluate the association between exposure variables and malformation in univariate analysis. To estimate the association between paternal exposure and malformation, we used the mother’s education level as the stratification variable. To control for the effect of potential confounding variables, unconditional logistic regression analysis was applied, initially including all variables with *p* < 0.20 in univariate analysis.. The final model included the variables with *p* < 0.05 and those considered important a priori according to the literature. The following variables were maintained in the final model: an interaction term between paternal pesticide application (yes/no) and maternal education level (< high school/high school or +), father currently farms (yes/no) and paternal past pesticide application (yes/no). The analyses were performed with the programs Epi Info version 7 (CDC,.Atlanta, GA, USA) and Stata (Stata 12, StataCorp LP, College Station, TX).

## Results

The study included 137 cases and 274 controls. Three case mothers and seven control mothers refused to participate in the study, but they were immediately replaced with subsequent mothers until the minimum sample size was achieved. The distribution of congenital malformations, deformations and chromosomal abnormalities among cases is in Table 1. Table 2 shows the sociodemographic characteristics of the children (sex, race/ethnicity, low birth weight) and their mothers (age group, race/ethnicity, marital status, education level and family income). In relation to the children, there were no statistically significant differences between cases and controls. Among the mothers, marital status differed significantly between cases and controls and being married was associated with congenital malformation (*p* = 0.033).

**Table 1:** Distribution of socio-demographic characteristics of cases and controls and of their mothers, Cuiabá, MT, 2011

Variables	Case		Control		
	*n*	(%)	*n*	(%)	*p*-value
Children					
Sex					
Male	85	62.0	170	62	-
Female	52	38.0	104	38.0	
Race/Color					
White	53	38.7	113	41.2	0.619
Other^a^	84	61.3	161	58.8	
Low birth weight					
No	107	78.1	224	81.7	0.454
Yes	30	21.9	50	18.3	
Mothers					
Age group					
< 18 and ≥ 38	09	8.8	31	11.3	0.425
18 to 37	125	91.2	243	88.7	
Race/ethnicity					
White	42	30.7	80	29.3	0.770
Brown and Black	95	69.3	193	70.7	
Marital status					
Married^b^	119	86.9	214	78.1	0.033
Single/Widowed	18	13.1	60	21.9	
Education level					
< High school	65	47.4	148	54.2	0.196
High school or +	72	52.6	125	45.8	
Family income per member^a^					
< 3 Min salaries	103	75.2	190	69.6	0.238
3 or + min salaries	34	24.8	83	30.4	

^a^Minimum salary = $545.00 (2011)

^b^Married or living with partner

**Table 2:** Odds ratio and 95 % confidence interval (95 % CI) of maternal^a^ and paternal^b^ pesticide exposure variables and congenital malformation, Cuiabá-MT −2011

Variables	Case		Control			
	*n*	(%)	*n*	(%)	OR (95 % CI)	*p*-value
Maternal exposure						
Residence						
Rural area	9	18	8	7.2	2.82 (1.02–7.82)	0.039
Urban area	41	82	103	92.8	1.00	
Lives close to crops sprayed with pesticides						
Yes	119	88.1	217	82.2	1.61 (0.88–3.03)	0.123
No	16	11.9	47	17.8	1.00	
House has garden/orchard						
Yes	41	29.9	61	22.4	1.47 (0.92–2.34)	0.098
No	96	70.1	211	77.6	1.00	
Uses pesticides at work						
Yes	11	21.6	17	15.3	1.52 (0.65–3.53)	0.329
No	40	78.4	94	84.7	1.00	
Paternal exposure						
Residence						
Rural area	32	24.4	56	22.4	1.11 (0.68–1.84)	0.656
Urban area	99	75.6	194	77.6	1.00	
Works on a farm						
Yes	114	86.4	206	78.9	1.69 (0.95–3.03)	0.073
No	18	13.6	55	21.1	1.00	
Applies pesticides						
Yes	17	65.4	24	40.7	2.75 (1.05–7.19)	0.036
No	09	34.6	35	59.3	1.00	

^a^Maternal exposure period defined as the 3 months before and the 3 months after conception

^b^Paternal exposure period defined as the 12 months that preceded conception

In univariate analysis (Table 3) the variables “mother living in rural areas” (OR = 2.82; 95 % CI 1.02–7.82) and “paternal past pesticide application” (OR = 2.75; 95 % CI 1.05–7.19) were significantly associated with congenital malformations. Moreover, we observed positive effect modification of the association between “father with past pesticide application” and congenital malformations when the mother had low evel of education (incomplete high school) (OR = 2.98; 95 % CI 1.25–7.11) (Table 4).

**Table 3:** Odds ratio and 95 % confidence interval (95 % CI) of paternal pesticide exposure up to 1 year before conception and occurrence of congenital malformations stratified by maternal education level, Cuiabá-MT - 2011

Variables	Case		Control		
	*n*	(%)	*n*	(%)	OR (95 % CI)
Occupation^a^					
Did not apply pesticides	58	84.1	103	85.8	1.00
Applied pesticides	11	15.9	17	14.2	0.87 (0.38–1.98)
Occupation^b^					
Did not apply pesticides	56	88.9	102	72.9	1.00
Applied pesticides	7	11.1	38	27.1	2.98 (1.25–7.11)

^a^Maternal education level = high school or more

^b^Maternal education level = incomplete high school

**Table 4:** Logistic regression, odds ratios, and 95 % confidence intervals (95 % CI) of variables associated with congenital malformations by cases and controls. Cuiabá-MT- 2011

Variables	OR	95 % CI	*p*-value
Paternal pesticide application^a^ * LMEL^b^	8.40	2.17–32.52	0.002
Father currently farms	4.65	1.03–20.98	0.045
Paternal past pesticide application^a^	4.15	1.24–13.66	0.021

Adjusted for number of prenatal visits, maternal age at delivery, and offspring’s birth weight

^a^12 months before conception

^b^ *LMEL* low maternal education level (< high school)

The final logistic regression model included the ‘interaction term’ between paternal past pesticide application and low maternal education level (OR = 8.40; 95 % CI 2.17–32.52) and the variables “father currently farming” (OR = 4.65; 95 % CI 1.03–20.98) and “paternal past pesticide application” (OR = 4.15; 95 % CI 1.24–13.66) (Table 4).

## Discussion

The study results suggest an association between parental pesticide exposure and offspring congenital malformations. Regarding paternal exposure, the same association was found between fathers who farm and fathers who do not farm but used pesticides on farms or elsewhere. These results corroborated studies that assessed parental pesticide exposure before conception and the occurrence of congenital nervous system malformations [2, 32, 33]. Recio et al. (2001) [29] reported that pesticides act on male fertility by changing sperm morphology and mobility and other semen components, increasing the likelihood of congenital malformations in the offspring of exposed males.

Mato Grosso uses much more pesticides than other Brazilian states. Oliveira et al. (2012) [34] studied environmental exposure to pesticides in the population of Mato Grosso and identified many municipalities with high-yield, highly mechanized monocultures with large-scale pesticide use. Another population-based study conducted in the same municipalities by some of the same authors also found an association between maternal pesticide exposure and congenital malformations [7].

Mothers who were married or lived with a partner were more likely to have offspring with congenital malformation in relation to those without a partner. Cavieres (2004) [35] found that the offspring of mothers who do the laundry of fathers exposed to pesticides are at greater risk of congenital malformations.

In parental exposure analysis, after stratifying for maternal education level, mothers with low educational attainment were more likely to have offspring with congenital malformations in comparison to those with high educational attainment. A possible explanation for these findings could be that a low level of education may result in the inadvertence in handling clothes and utensils contaminated by pesticides with consequent greater risk of exposure [35].

Living in a rural area was associated with congenitally malformed offspring regardless of paternal exposure to pesticides, corroborating Bell et al. (2001) [36]. In Mato Grosso, Belo et al. (2012) [6] detected higher concentrations of organochlorides in the blood and urine of people living in the rural areas of municipalities with intense pesticide use than in people living in the urban areas of the same municipalities, confirming the higher contamination of rural areas.

In the present study, having few prenatal visits was not associated with congenital malformations, contrary to the findings of Pinto & Nascimento (2007) [37] and Guerra et al. (2008) [38], where the number of prenatal visits was inversely associated with the presence of congenital malformations. However, it is important to note that some family health teams and primary care units in Mato Grosso have received guidance on the risks of exposure to pesticides and its deleterious health effects. Proper prenatal medical assistance may confer a protective effect for the occurrence of birth defects [12].

The study has some limitations. In order to assess the association between pesticide exposure and malformations, we had to lump all kinds of malformations together, due to the small sample size. Unfortunately, this same problem made it impossible to evaluate individual associations between a specific malformation and pesticides. Although this procedure could have introduced an information bias in the study, we believe that it would lead toward the null hypothesis, as some malformations would be related to pesticide exposure but not others. Pesticides are demonstrably one of the most toxic agents for use in local agriculture. However, one cannot exclude the influence of other likely environmental contaminants such as heavy metals used in adjacent mines or even other agrochemicals used in regional agriculture. Also, instead of a case–control, the ideal study would have being a longitudinal study to identify environmental exposures of prospective parents and follow them during pregnancy and birth in order to identify live born children with malformations. However, it is difficult to conduct this type of epidemiological study when the outcome disease is a rare event. The interviewers were well trained and observed closely during the interviews to minimize observation biases. Regarding recall bias, mothers of congenitally malformed children may have remembered pesticide exposure better than those with children not affected. However, all controls were hospitalized children and, as so, they had a health condition that could influence their mothers information as well. Mothers of younger children may also have remembered pesticide exposure better than those of the older ones, but this bias would have affected cases and controls, as the age distribution of both groups was similar. Also only cases diagnosed at birth could have been included in the study, but given the small number of congenitally malformed newborns, this option would prevent the attainment of the minimum sample size. We did not exclude the possibility of having excluded children with different pesticide exposure profiles, like those with congenital malformations that were not hospitalized or those that, due to more severe malformations, were dead before the onset of the study. Cases and controls were matched for gender, because of the different endocrine disruptions caused by pesticides in male and female fetuses [9, 39]. However, these effects could have been controlled during multivariate analysis. Another limitation is that it was not possible to confirm the diagnosis of malformations by the detection of phenotypic abnormalities as the study had no funding provision for this. Also, the confidence intervals of the odds ratios from the final logistic regression model showed a wide range, probably due to the limited number of subjects in some strata. Nevertheless, the observed associations are strong and statistically significant and so, they could not be ignored.

This study is the first to evaluate the association of paternal and maternal exposure to pesticides on the occurrence of birth defects in the Brazilian Legal Amazon, region where monoculture crops use more pesticides in all the world [15]. It is also the first analytical study conduct in this area with exposure and outcome data collected and analyzed at the individual level, thus demonstrating the statistical association of those two variables among the population of Mato Grosso, Brazil.

It is expected that the study results may have the attention of researchers and policy makers in order to promote public health policies for the protection of these populations and the reduction of the use of “uncontrolled” pesticides in plantations.

## Conclusion

The study results show that both maternal and paternal pesticide exposure are associated with congenital malformations. Additionally, children whose fathers were exposed to pesticides and whose mothers have low education level are more susceptible to congenital malformations.

## Abbreviations

CI, confidence interval; CLASCM, collaborative latin american study on congenital malformation; ICD-10, tenth international classification of diseases; OR, odds ratio
